# A lot of variation and asymmetry in the white patches of male capercaillie tails, but no association with mate choice

**DOI:** 10.1002/ece3.73945

**Published:** 2026-07-01

**Authors:** Markku Milonoff, Antti Paasivaara

**Affiliations:** ^1^ Department of Forest Sciences University of Helsinki Helsinki Finland; ^2^ Natural Resources Institute Finland Oulu Finland

## Abstract

Male western capercaillies ( *Tetrao urogallus* ) exhibit conspicuous white patches on their elongated black tails. Since males display their tails during courtship to females, and since there are significant individual differences in the size and pattern of these patches among males, these traits could be subject to sexual selection and influence mate choice. The aim of this study was to investigate the significance of the amount and symmetry of these white patches in female choice. The study was based on game camera images collected from 13 display grounds in Southern Finland over a period of 9 years (17 lek‐years and 85 males). The results showed no evidence that the extent of white patches on the tails or their asymmetry affected the likelihood of males to mate. There was no difference whether asymmetry was measured as the absolute difference in the amount of white in individual tail feathers or as relative differences. Nor did the intra‐lek rank order of these traits among males better explain the distribution of matings. It appears that, despite their visibility and considerable individual variation, the tail patterning in males is not a target of female choice, and mate selection is based on other traits. Actually, the significance of the white patches may be to facilitate individual recognition. Capercaillie display is most intense at dawn, when the white markings stand out particularly clearly and can enable females and other males to identify individuals even from a distance.

## Graphical abstract

White patches of male capercaillie tails vary greatly in coverage and symmetry, but the amount of white or the symmetry of the patches was not associated with mate choice. Female choice does not appear to be based on the white patterns on the tail. Highly visible at dawn, the patches may instead facilitate individual identification.

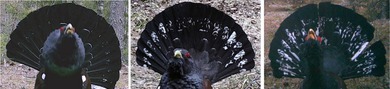

## 1 Introduction

Numerous bird species exhibit sexual dimorphism, which is manifested in differences of both plumage and structural characteristics (Andersson 1994; Cuervo and Møller 1999; Møller 2021). Dimorphic feathers used for ornamentation in male birds are considered to have evolved and be maintained through sexual selection as secondary sexual characteristics. Since the producing and maintenance of these traits incur costs that may manifest, for example, as increased predation risk or physiological costs associated with suppressed immune system (review in Cuervo and Møller 2000), they should have a positive impact on the reproductive success of the male. Research has shown that these characteristics are at least largely influenced by female mate preference and they only seldom have significance in male–male competition (reviews in Andersson 1994; Møller 1994). However, several studies have not found that males with the extreme ornaments have a selective advantage in mate choice (Gibson and Bradbury 1987; McDonald 1989; Pruett‐Jones and Pruett‐Jones 1990; Zuk et al. 1990; Wittzell 1991; Savalli 1994; Liu et al. 2018) and other theories have also been proposed to explain these exaggerated traits (review in Zhou et al. 2023).

The elongated tails and tail feathers of males are one of the most common sexual traits of bird plumage (review in Zhou et al. 2023). Tails have primarily been studied as morphometric structures that can be measured. The length of males' tails has been shown to influence females' mate choice and males' number of offspring, but not in all species (review in Cuervo and Møller 1999; see also for example, Savalli 1994; Rintamäki et al. 2001; McFarlane et al. 2010; Costanzo et al. 2017; Liu et al. 2018). Tails often have also conspicuous colours and patterns that are displayed to females. Overall, the significance of plumage colours and patterns has been less explored (but see e.g., Safran and McGraw 2004; Siitari et al. 2007; Jiguet and Bretagnolle 2014), likely due to the methodological restrictions and difficulties in measuring them (Pérez‐Rodríguez et al. 2017). However, the presence of sexually dimorphic bright colours and complex patterns in tails strongly suggests that they play a role in mate choice, especially when there are documented examples that exaggerated tails may have detrimental effects on individuals (Møller and Nielsen 1997; Saino and Møller 1996; Saino et al. 1997). Coloration may amplify the negative effects, although some patterns may, on the other hand, mitigate these effects. Certain studies have provided evidence for the effect of tail colours and patterns on males' success (Höglund et al. 1990, 1994; Petrie et al. 1991; Kose et al. 1999; Loyau et al. 2005; Stein and Uy 2006; Soulsbury et al. 2016; Costanzo et al. 2017; Minias et al. 2018), but a small number of studies have reached the opposite conclusion (Sæther et al. 2000; Takahashi et al. 2008 and references therein). A large proportion of these tail colour studies are related to white tail patches, that have been suggested to have special significance as quality signals because they are prone to wear, preferred by feather mites, and distinct in low light (Hasson 1991; Fitzpatrick 1998; Kose et al. 1999).

Another characteristic that has been studied in tail feathers is fluctuating asymmetry (FA). FA refers to small, random deviations from symmetry in bilaterally symmetrical characters (e.g., Møller and Swaddle 1997). These deviations are believed to reflect the individual's ability to control development or resist stress. The causes of FA can be both genetic (e.g., mutation) and environmental (e.g., food deficiency, parasites). FA in secondary sexual characters is found to be larger than that of ordinary morphological characteristic, suggesting that ornaments are more susceptible to disruption of developmental homeostasis or stress (Cuervo and Møller 1999; but see e.g., Björksten et al. 2000). Many studies indicate that females choose their partners based on symmetry of secondary sexual characteristics (review in Møller and Thornhill 1998; but see Simmons et al. 1999). These features also include tail feathers (review in Cuervo and Møller 1999; see also Costanzo et al. 2017). Studies that have examined the asymmetry of tails have used differences in the lengths of tail feathers to measure asymmetry. However, to our knowledge, the asymmetry of tail coloration or patterning has been studied only in populations of peacocks with conflicting results (see Takahashi et al. 2008). Nonetheless, the asymmetry of tail patterning may be more easily and clearly observed in mate selection than small differences in feather length. The development of patterning may also be more sensitive and revealing to developmental disorders. The impact of certain environmental factors may be more visible in patterning (e.g., external parasites Kose et al. 1999 and deficiency of certain nutrients Hill and Johnson 2012), but the type of patterning can affect how sensitive it is (e.g., abrasion in the middle of feathers compared to the tips of feathers).

Western Capercaillie ( *Tetrao urogallus* , hereafter Capercaillie) is a large sexually dimorphic gallinaceous grouse species inhabiting coniferous forests. Dark‐coloured males gather in lekking sites and display largely in the early morning twilight. They have clearly visible white patches on their long tail, with the amount and placement varying greatly between individuals. Males display their fanned tail to females, which choose their mating partners and express their readiness to mate through squatting. The purpose of this study was to examine whether the white patterns on capercaillie males' tail are associated with female mate choice. Both the amount of white in the tail feathers and the symmetry of the white patterns distribution on the tail were examined. More specifically, we investigated whether males that received matings generally have more or less white in their tails compared to those that did not receive matings, and whether the white patches are more symmetrically distributed on either side of the tail in mated compared to unmated males.

## 2 Material and Methods

### 2.1 Study Species and Study Area

The study was carried out during 2014–2023 in southern Finland, around Evo (61°13’N, 25°06’E) and Valkeakoski (61°14’N, 24°12’E). In the leks and years included in this study, the minimum number of territorial males per year ranged from 2 to 16 (mean = 5.0 ± 2.83, *N* = 17). In total, there were 85 males. Leks were located in forests over 40 years old, most often at the edges of bogs.

Over two‐years‐old males have well‐defined and rather large display territories. The spring display extends for approximately three months, but females visit the lek for only a short period of a few weeks (e.g., Hjorth 1970; Wegge and Larsen 1987; Storch 1997). Females choose their mating partners and express their readiness to mate through squatting. Mating hardly ever occur immediately; instead, squatting females circle around the male on his display mound even for hours, often over several consecutive mornings before the male mates with them. Mating is distributed very unevenly, with one or only a few males taking part in the mating (Lumsden 1961; Müller 1979; Moss 1980; this study). Typically, a group of several females gathers around these males, and it is apparent which male is favoured by the females.

**FIGURE 1:**
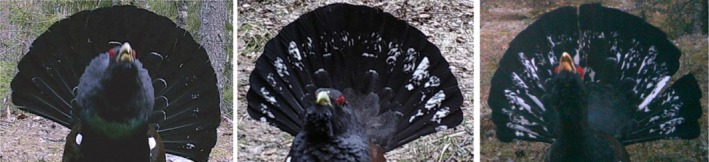
Examples of different capercaillie males' tails. Left and right are not the extremes of variation (pictures showing the entire tail in the same image are rare). There may also be more asymmetry than in these pictures. The male on the right has earlier lost some tail feathers and at least one of the new feathers, which has a lot of white in it, is still growing. All the males in the pictures successfully mated that year.

The plumage of male capercaillies consists mainly of black, dark brown, and grey colours. The dark breast has shades of blue and green. Combs are red. The long tail feathers are black (rarely some grey in the two centremost) with white patterns which are highly variable between individuals (see Figure 1). There is white also on the underside of the wings (not seen when on ground) and a spot at the bend of the wings, as well as irregularly on the stomach and under the tail. During courtship, males actively display their fully spread tail to females. The white patterns of the black tail are clearly visible even in low light.

### 2.2 Identification of Individual Males From Game Camera Images

Individual identification was based on images captured by a game camera. Displaying males at a particular lek could be identified from close‐distance images on the basis of white patterning of spread tail and undertail coverts. Additionally, the white patterning on the stomach, white feathers and spots in unusual locations, as well as plumage damages, could be of help in the identification process.

Game cameras were placed in the core areas of male lekking territories, where males spend most of their display time and where females also gather and where most of the mating occurs. These locations are typically small mounds or slightly more open areas. Core areas were found on the basis of observations, snow tracks, and abundant droppings, in particular. The core areas are highly persistent, and successive males that dominate the same territory usually use the same mounds, so the researcher's previous experience also helps in locating them.

The cameras were placed usually at a height of 50–100 cm from the ground and they were programmed to capture series of photos without delay when motion was detected. Rarely, when the core area was larger, the camera was programmed to take time‐lapse photos (usually at one‐minute intervals), allowing for coverage of a broader area. The goal was to obtain images of all territorial males on a display ground. During the weeks when females visited the males' territories, all core areas were photographed, unless it was already known which areas the females concentrated on. The number of cameras was not sufficient to cover all core areas of the display grounds in every year, and only those display grounds in a certain year which fulfilled this criterion are included in the data (17 lek‐years and 85 males). Two observations were accepted from the same lek if there was a minimum of 5 years between them. Territorial males remain at the same lek site throughout their lifetime, and due to the long time span involved, the likelihood of the same individual appearing in the dataset more than once is very low. Nevertheless, the characteristics of males from the same lek were compared, and no evidence suggested the presence of the same individuals more than once. Males that were observed mating or were surrounded by frequently soliciting females were interpreted as having received mating (20/85 males). If there were two males mating at the same lek (three leks), both were included in the data.

### 2.3 Estimation of the Amount of White and the Asymmetry of Patches in the Tail

**FIGURE 2:**
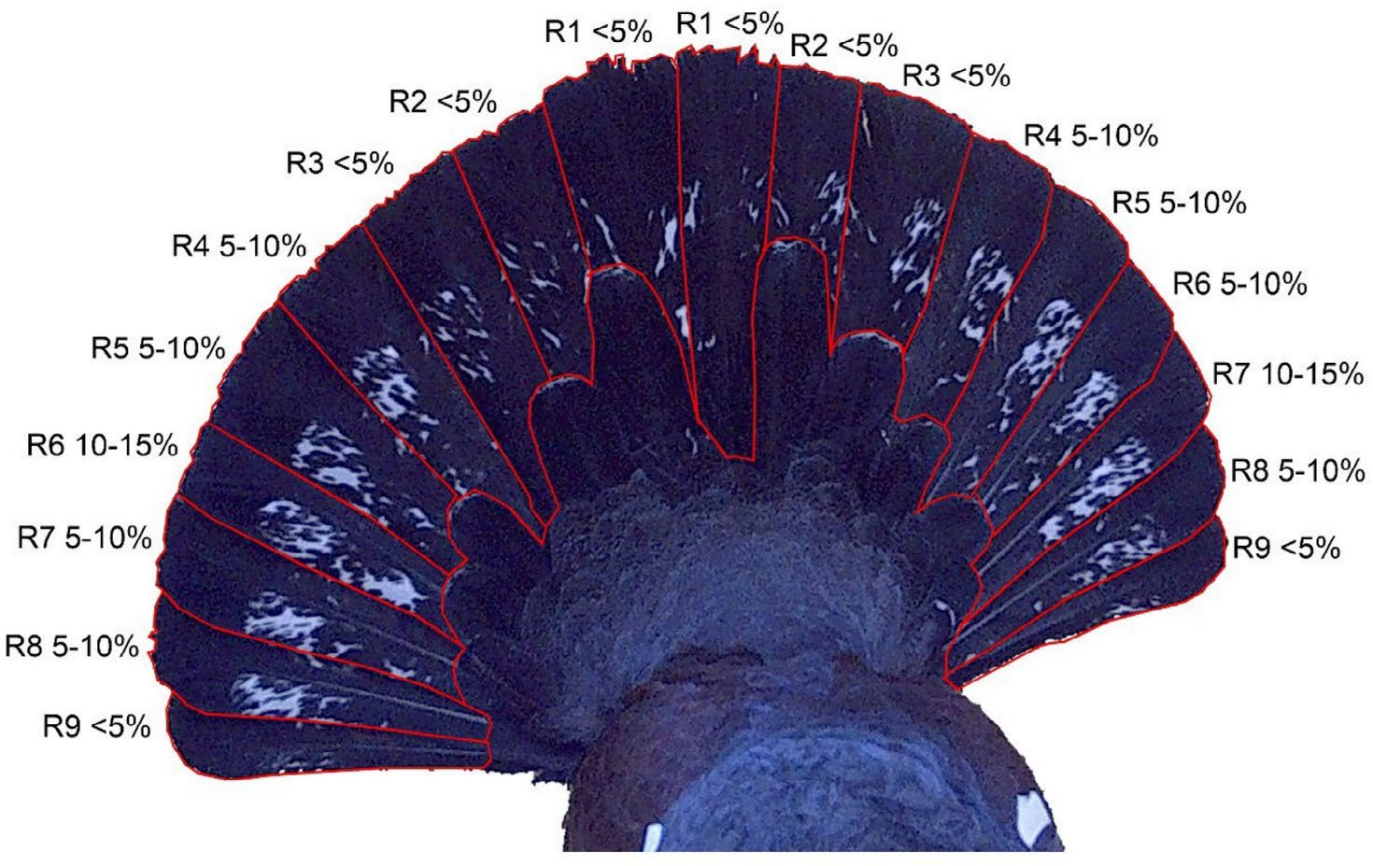
Example image of tail feathers and a visual estimate of the amount of white in the feathers. The total amount of white in the tail is 105 pp. (= the sum of the category means) or 5.8% per tail feather. The amount of absolute asymmetry is 10 pp. (= the sum of the absolute values of the difference of category means for the tail feather pairs). The amount of relative asymmetry is 0.095 (= the sum of the absolute values of the difference of category means for the tail feather pairs divided by the sum of the cathegory means). In this particular tail, the amount of white is fairly average, and there is little asymmetry.

The estimation of the amount of white in the tail was based on the percentage of white colour area in that part of the tail feathers that was visible under the covert feathers (Figure 2). The assessment was made from a fully spread tail (angle between outer tail feathers at least 170 degrees) and the photo had to be taken almost directly from the front. In this position, the entire outer half and part of the inner half of a tail feather are visible underneath the feather on top. The assessment was conducted from a computer screen, and the image was enlarged using image editing software so that the tail covered a large part of the display. Capercaillie has nine tail feathers on each side of the tail. The visible part of each tail feather was cropped, primarily based on the shape of the feather tip, the visible edges of the feathers and the edges of the white patches. The proportion of white colour was assessed separately for each feather using the following scale: 0% (no white), less than 5% white, 5%–10% white, 10%–15% white, 15%–20% white, etc. If a tail feather was missing, it was assigned a value of 0% (two males from the same lek, one of whom mated, see below). To describe the overall amount of white in the tail feathers, the sum of average values for each category (0%, 2.5%, 7.5%, 12.5%, 17.5%, etc.) was used:

$$\sum_{i=1}^{9}\left(L_{i}+R_{i}\right)$$

where L and R are the same tail feather on the left and right sides.

In only a few cases, the whole tail was visible in one photo because the head usually covered part of it. However, the hidden part could often be seen in another photo. Altogether, the assessment of white areas on the tail could be conducted for 85 individuals for 17 lek‐years, when the distribution of mating among males was also known (including those leks where two lek‐years were accepted).

Fluctuating asymmetry was defined in two ways: 1. Absolute difference in the amount of white = sum of absolute values of differences in the percentage points of white colour in individual tail feathers (left and right):

$$\sum_{i=1}^{9}|L_{i}-R_{i}|$$

where L and R are the same tail feather on the left and right sides.

2. Relative difference in the amount of white = sum of absolute values of differences in the percentage points of white colour in individual tail feathers divided by the sum of percentage points of white in all tail feathers combined:

$$\sum_{i=1}^{9}|L_{i}-R_{i}|÷\sum_{i=1}^{9}\left(L_{i}+R_{i}\right)$$

where L and R are the same tail feather on the left and right sides.

Absolute values were used because directional measures of the amount of white and symmetry (± left/right) often cancel each other out, making the calculation of symmetry impossible when zero values are involved. These definitions do not consider differences in the shape of patterns, but they are certainly related to the symmetry of the patterns (see below).

### 2.4 Repeatability, Reliability and Validity of Estimates

The assessment of all tail images was conducted by the same person. The repeatability of visual white colour coverage estimates was assessed by measuring images of 10 tails two times without the knowledge of the results obtained the first time (time between measurements over 2 weeks). Repeatability was high (*r*_s_ = 0.961, *N* = 180 feathers of 10 tails, *p* < 0.001), suggesting that measurements were precise enough for analyses.

The reliability of coverage estimates and asymmetry indexes were tested utilising ImageJ software (rsbweb.nih.gov) to determine the percentage coverage of white patches in 10 tail images, in which the tail was fully visible in one image. When using the software, the image was first converted into an 8‐bit grayscale image. Subsequently, the image was adjusted with an auto‐threshold method, that best distinguished white areas from black feather background. Different tail feathers were cropped from the image, and the programme determined the percentage of areas with white patches from the total feather area. The percentage of white patches in the entire tail's area was also obtained. In some analyses of these tails (e.g., due to lighting condition) two different images (but the same auto‐threshold method) from the same series of images were used for different sides of the tail. The results of the ImageJ application were compared to the visually estimated values for the same tails (Table 1). Although the ratings differed quite a bit for some tails (e.g., the software did not consider all small spots, the classification impact of visual assessment, lighting conditions), the rank correlation between visual indexes and the values provided by the ImageJ application was high. Thus, the visual assessments adequately represent the quantity of white spots and the asymmetry of their distribution on the tails, allowing the tails to be ordered based on these visual indexes. Using the ImageJ application for this purpose is very laborious (manually outlining feathers is time‐consuming) and it cannot be used for all images of males (reflections and shadows affect the result, for example, the rachis, although black, often appears as a white area in the image), although a visual estimation of the proportion of white patterns in their tails can still be made.

**TABLE 1:** Spearman rank correlations between visual estimates of white patches in ten tail images and values measured with ImageJ software as well as rank evaluations of symmetry by 11 raters (average). The ranks obtained using different methods were compared using Spearman's rank correlation coefficient.

	ImageJ		Raters		N
	*r*_s_	*p*	*r*_*s*_	*p*	
Area of white patches (visual estimates)	0.97	< 0.001***			10
Relative amount of asymmetry in the white patches (visual estimates)	0.83	0.003**	0.80	0.005**	10
Absolute amount of asymmetry in the white patches (visual estimates)	0.79	0.010**	0.56	0.096	10

* *p* < 0.05.

** *p* < 0.01.

*** *p* < 0.001, two‐tailed.

The impression of asymmetry in the white patterns on the tails is not only influenced by differences in coverage but also differences in patch shapes. The relationship between coverage‐based indexes and the perceived asymmetry of the tails was tested by asking 11 people to rank the same 10 tails on the basis of their symmetry. The evaluators were able to rank especially the most symmetrical and asymmetrical tails consistently (Table 1). The evaluators' rankings were more similar to the relative amount of asymmetry than to the absolute amount of asymmetry, probably because the evaluators were asked to pay attention to the relative asymmetry. The correlation between the relative asymmetry index and the evaluators' rankings was high (Kendall's coefficient of concordance W = 0.64, *p* < 0.001, *N* = 11), indicating that the index also represents the asymmetry impression of the tails. Evidently, the indexes can replicate the situation in which female capercaillies could be ranking the males at a lek site based on the amount of white colour and the symmetry of the white spots on their tails.

We also calculated the coefficient of variation (SD divided by mean) to compare the degree of variability in the characteristics with the typical variation of sexual ornaments. Ornamental characteristics typically exhibit high variability. The variation among individuals in these characteristics has often been found to be large (e.g., Cuervo and Møller 1999).

### 2.5 Statistical Analyses

We used generalised linear mixed models (glmmTMBs) with a binomial error distribution to study whether the amount of white on the tail, the asymmetry of the patches, or the interaction between these variables affects the likelihood of males mating. To test as many mate‐choice criteria as possible, we conducted the testing using the direct measures of white coloration and asymmetry, and, separately, the within‐lek rank order of males (tied ranks) with respect to these traits. For asymmetry, we also used both the absolute and relative measures of asymmetry, resulting in a total of four different models (see Table 3). The explanatory variables were centred and scaled (z‐scores) (Schielzeth 2010) and the R performance package was used to assess multicollinearity (e.g., all variance inflation factor VIF values smaller than 1.49). We fitted a null model, which contains only an intercept, and models including the explanatory variables and used a likelihood‐ratio test to assess the relative goodness of fit of the models including the explanatory variables compared with the null model. Lek was included as a random factor in all models (also in null model), because females choose their mates from among the males on a given lek. To ensure that the specified 0% white for missing tail feathers does not bias the results, we conducted the same analyses excluding the lek where such birds were present.

All statistical analyses were performed in R 4.5.0 (R Development Core Team 2025) using the performance package version 0.15.3 (Lüdecke 2019) and glmmTMB package's (version 1.1.14) glmer function (Brooks 2017).

## 3 Results

**FIGURE 3:**
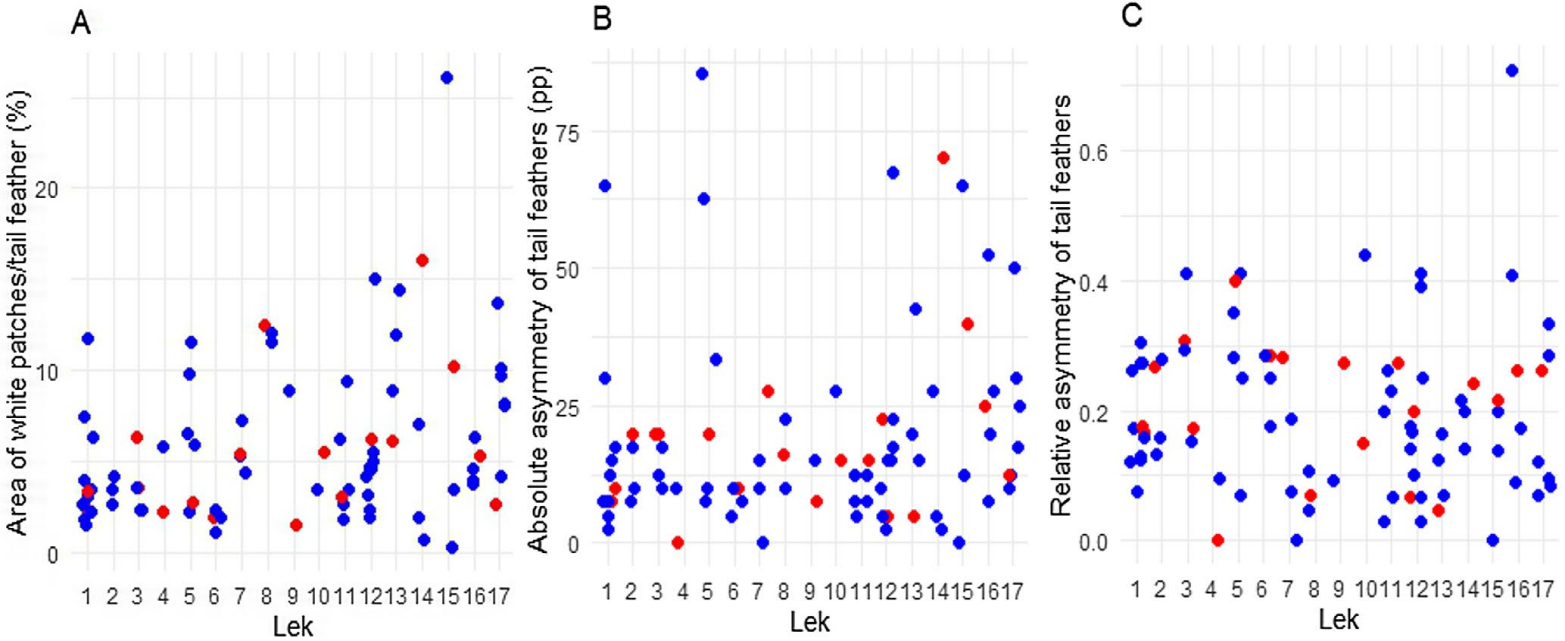
Estimates of coverage of white patches and asymmetry of patches in tails of mated (red dots) and not mated (blue dots) capercaillie males in different leks.

Estimates of coverage of white patches and asymmetry of patches in tails of mated, not mated and all capercaillie males are presented in Figure 3 and Table 2. The variation in these characteristics among individuals was large (coefficient of variation 63%–92%).

**TABLE 2:** Estimates of area of white patches and asymmetry of patches in tails of mated, not mated and all Capercaillie males. Area of white patches in a feather is the average of all feathers in the tail. Absolute amount of asymmetry is the average of differences in the percentage points (pp) of white colour between individual left‐ and right‐side tail feathers (9 pairs). Relative amount of asymmetry is the sum of differences in the percentage points of white colour in individual left‐ and right‐side tail feather pairs divided by the sum of percentage points of white in all tail feathers combined. Sample sizes are: 20 for mated males, 65 for not mated males and 85 for all males in all cases.

	Average area of white patches in a tail feather (visual estimate)	Relative amount of asymmetry in the white patches of tail feather pairs (visual estimate)	Absolute amount of asymmetry in the white patches of tail feather pairs (visual estimate)
Mean ± SD			
Mated males	5.3 ± 3.7 (%)	0.21 ± 0.10	2.0 ± 1.7 (pp)
Not mated males	5.9 ± 4.4 (%)	0.19 ± 0.13	2.1 ± 2.0 (pp)
All males	5.7 ± 4.3 (%)	0.20 ± 0.12	2.1 ± 1.9 (pp)
Minimum—Median—Maximum			
Mated males	1.5–4.2–16.1 (%)	0.0–0.23–0.40	0.0–1.7–7.6 (pp)
Not mated males	0.3–4.4–26.2 (%)	0.0–0.17–0.72	0.0–1.4–9.5 (pp)
All males	0.3–4.2–26.2 (%)	0.0–0.18–0.72	0.0–1.4–9.5 (pp)
Coefficient of variation (%)			
Mated males	71	49	81
Not mated males	76	66	96
All males	75	62	93

There was no evidence that the coverage of white patches in tails or patch asymmetry affects the likelihood of capercaillie males to mate (Table 3). There was no difference whether asymmetry was measured as absolute difference in the amount of white in individual tail feathers or as relative differences. Nor did the males' intra‐lek rank order of these traits better explain the distribution of matings. None of the four models fitted the data better than the null model, which included only a constant: the likelihood ratio test *p*‐values were all larger than 0.837 (Table 3). The results remained unchanged, even when the analyses were performed without the lek containing birds with missing tail feathers (likelihood ratio test *p*‐values all larger than 0.670). In addition to linear models, we tested four models that included all explanatory variables also raised to the second power. These models did not fit the data better than the null model either (likelihood ratio test *p*‐values all larger than 0.264).

**TABLE 3:** Generalised linear mixed models (GLMMs) for the effect of white patches and their asymmetry, based on both direct measurements and within‐lek rank order the males, on the likelihood of capercaillie males to mate. The likelihood ratio test assesses the model's performance relative to the null model, which includes only a constant. Lek is treated as a random variable in all models.

	Conditional model				Likelihood ratio test	
	*β*	SE	*z*	*p*	Deviance	*p*
**Direct measures** Model 1						
Amount of white	−0.220	0.391	−0.562	0.574	−46.159	0.933
Absolute amount of asymmetry	0.137	0.387	0.355	0.722		
Amount of white × absolute amount of asymmetry	−0.024	0.231	−0.105	0.722		
Model 2						
Amount of white	−0.128	0.286	−0.446	0.655	−46.164	0.936
Relative amount of asymmetry	0.086	0.265	0.324	0.746		
Amount of white × relative amount of asymmetry	0.070	0.353	0.198	0.843		
**Within‐lek rank order of males** Model 3						
Rank in amount of white	−0.281	0.414	−0.679	0.497	−45.949	0.837
Rank in absolute amount of asymmetry	0.223	0.374	0.597	0.550		
Rank in amount of white × rank in absolute amount of asymmetry	−0.059	0.226	−0.262	0.793		
Model 4						
Rank in amount of white	−0.260	0.342	−0.760	0.447	−46.064	0.891
Rank in relative amount of asymmetry	0.045	0.287	0.158	0.875		
Rank in amount of white × rank in relative amount of asymmetry	0.070	0.216	0.323	0.747		

## 4 Discussion

### 4.1 Conspicuous and Highly Variable Patches, but no Association With Mate Choice

The white markings on the tails of male capercaillie vary greatly among individuals, and during courtship they are prominently displayed to females by males spreading their tails. These markings are especially visible in the dim light of early morning when the courtship display is typically at its most intense. Therefore, it could be strongly assumed that these patches are ornaments subject to sexual selection and influence female mate choice. However, the amount of white on the tail was not associated with mate choice. The results of this study are thus different from the majority of previous research with other species, in which the patterning of tails has been found to influence female mate choice (Höglund et al. 1990, 1994; Petrie et al. 1991; Kose et al. 1999; Loyau et al. 2005; Stein and Uy 2006; Costanzo et al. 2017; Minias et al. 2018), and adds to fewer studies that have not found an effect (Sæther et al. 2000; Takahashi et al. 2008 and references therein).

Fluctuating asymmetry in coverage of white spots on the tail feathers was neither associated with mate choice, although it also varied a lot (relative amount of asymmetry 0–0.72 and absolute amount of asymmetry 0–9.5 pp., Table 2) and although there was significant individual variation (relative amount of asymmetry 62% and absolute amount of asymmetry 93%, Table 2). FA indexes measured across different species are highly variable (see Cuervo and Møller 1999), but the variations reported in this study are towards the higher end, although even larger values have been reported (e.g., for number of eyespots in tails of peacock even over 100%, Takahashi et al. 2008). To our knowledge, the only species, in which the relationship between tail pattern asymmetry and mate choice has previously been studied, is the peacock (see Takahashi et al. 2008 and references therein). Results have been controversial, and this study is consistent with those peacock studies where no correlation was found.

### 4.2 Why Was no Association Found?

Interpreting negative results in studies of this kind can be challenging due to methodological difficulties. The precision of measurement may not be sufficient to capture the effect or the variation of the trait is small (see Cuervo & Takahashi et al. 2008). Because the coverage of the white patterns on the capercaillie males' tails varied so much (from almost zero to one fourth, Table 2), there should not be such a problem in the data. There would be even more variation if only the sector of the tail feathers, in which the white patches are located, is considered (about one third of the tail feathers from the tip towards the base do not have patches). Individual variation was also huge (75%, Table 2) compared to morphometrical measures of ornament traits and tails in other species where association with mate choice has been found (CV usually less than 20%, in Cuervo and Møller 1999). Also, variation in measured FA indexes in tail feather lengths across different species is generally smaller (see Cuervo and Møller 1999). Although conspicuous plumage colours are generally highly variable (Delhey et al. 2017), the variation in the amount of white in male capercaillie tail feathers was clearly greater than the few values we found in the literature (about 19% for white in great snipe tail feathers Höglund et al. 1990, about 5% for number of eyespots in peacock tail Takahashi et al. 2008). For capercaillie females the white markings in the spread tails of males are also extremely visible against the black background of the feather. The large differences in white patterns of male capercaillie tails are surely more easily detectable by females than small differences in tail feather lengths. Hence, in this study, a possible positive (or negative) association between the amount of white or FA and mate choice should have easily emerged.

The white of feathers is a structural colour and the black is a melanin‐based colour, not incurring costs associated with scarce nutrients from the diet like carotenoid‐based colours (e.g., Prum 2006). However, dark and white feather patterns can still be the target of sexual selection. White tail spots may be reliable signals or amplifiers of signals (Hasson 1991; Fitzpatrick 1998; Kose et al. 1999). White tail patches have even been suggested to have special significance as quality signals because without the structural support of melanin, they are prone to wear. The white patches on the tail feathers of male capercaillies are mostly in the middle of the barbs (excluding the tips of the tail feathers of 1‐year‐old males) and are not as prone to wearing down as the edges of the feathers. Feather lice also prefer the white areas of the feathers, and poorly maintained white areas can indicate a high number of external parasites, poor condition, and weak immunocompetence (Kose et al. 1999). However, there does not appear to be any noticeable wearing or fraying in the white patches on capercaillie tail feathers (own observations from shed feathers).

In this study, the white patterning on the tails of male capercaillies was not associated with female mate choice, but anyway there are still conspicuous white patches on the tails and significant variation between individuals. Males also actively display their spread tail to females. There may be some other characteristics on the tails that influence mate choice (e.g., length) that are not related to the white patterns. White spots could potentially amplify this trait in certain situations, but they do not have a consistent association with the characteristic. In this study, potential patterning visible in ultraviolet light was not considered, but on the other hand Galliformes have poor UV vision (Hart 2001; but see Siitari et al. 2002). In addition, the feather colours black and white do not most likely belong to ultraviolet colours (blues, greens, yellows and reds; Burkhardt 1989).

Female capercaillies probably exhibit mate fidelity (see Milonoff 1995 and references therein, own observations), like, for example, black grouse (Rintamäki et al. 1995), and only change male when their previous partner is not available. Therefore, the most important mating season for mate choice is when the female chooses a male for the first time (or possibly even previous seasons when she observed males). Later changes in mate choice criteria (such as losing tail feathers) may not affect the choice. Mate choice copying (Milonoff 1995; for black grouse see Höglund et al. 1995) may further weaken the connection between studied traits and mate choice. After all, if these traits are important, successful males should, on average, still have a particular type of white tail feather pattern.

### 4.3 Possible Other Functions for White Patches on the Tail

One possibility is that the white patterns on the tail may have evolved for other reasons than sexual selection. However, white patterns are hardly visible when the tail is tucked in, and grouse keep their tails tucked in when normally moving on the ground or perched in a tree. In addition to display, the white patches are particularly visible when a male spreads its tail as it takes off and also somewhat visible when guiding its flight with its tail. The white patches could possibly confuse predators when males are fleeing and taking off into flight. However, not all males have these spots, and this alone does not explain the large individual variation in patterns.

One clear feature of the white spots on male capercaillie tails is that they are especially visible in the early morning twilight, when the courtship is often at its most intense. Females observe males partly from a distance from the branches of trees and distinguishing dark males from each other on the basis of appearance could be difficult without the white patterns on their tails. Identifying individuals from a distance could also be important for males, allowing them to avoid unnecessary encounters and confrontations. If reliable identification of males based on sound is not possible, the varying patterns on their tails may have evolved to enable individual recognition. Especially large inter‐individual variation facilitates identification (Candolin 2003; Dale 2006).

In order to function as a reliable quality signal or unique identifying characteristics, traits should be fairly stable (Andersson 1994). The white patterns on the tails of capercaillie males are typically very stable within the same mating season. The tail features are rather similar even between years, but slight changes occur (Catusse 1993; Flor 2023: own observations). However, large changes happen when a male has lost some of its tail feathers. Such situations are quite common, and one can see on display grounds males that have lost almost all their tail feathers. In such cases, mate choice and male identification cannot be based solely on tail patterns. Sometimes, the tail feathers that replace lost feathers (and which may be growing during the mating season) are very different from the original feathers and often have a lot of white. This change may be due to damage to the feather follicle when the feather was torn off. Such changes can be permanent over moults and noticeable on the tail for several years (own observations). Because of these changes, mate choice and individual identification based entirely on the tail would be unreliable. Also, these unique tail feathers usually increase the asymmetry of white patches significantly and make asymmetry alone an unstable target for selection. The intact tail feathers could, however, indicate the viability of an individual, as seems to be typical for successful male black grouse (Alatalo et al. 1991). Nevertheless, male capercaillies, who have lost tail feathers or have tail feathers of different lengths or abnormal white feathers as a result of previous feather loss, may still be able to mate (own observations). On the other hand, exceptional tail feathers make individual identification easier.

## 5 Conclusions

In this study, despite the high individual variation, no association was found between mate choice and the amount of white in tail feathers or the asymmetry of white patches. It is possible that confounding factors such as male age, mate fidelity, and mate copying may be masking the connection, preventing it from being revealed through these research methods. Overall, the characteristics of a tail composed of relatively few feathers may not always, due to the rather common loss of tail feathers and differences between lost and replacing feathers, be a reliable and consistent indicator of an individual's quality. Another possibility is that the variation does not directly impact mate choice but rather allows males to be recognised by females or other males, especially in the early morning dusk.

## Author Contributions

**Markku Milonoff:** conceptualization (lead), investigation (lead), writing – original draft (lead), writing – review and editing (lead). **Antti Paasivaara:** data curation (equal), formal analysis (lead), writing – review and editing (supporting).

## Conflicts of Interest

The authors declare no conflicts of interest.

## Supporting information


**Appendix S1:** Average coverage of white patches in a tail feather, relative as well as absolute amount of asymmetry in the white patches of tail feather pairs in 10 tail images (estimated visually and measured with ImageJ software) and average symmetry rank of 11 raters for the same tails. See text for more details.

**Data S1:** ece373945‐sup‐0002‐DataS1.xlsx.

## Data Availability

All the required data are uploaded as Appendix S1.

## References

[ece373945-bib-0001] Alatalo, R. , J. Höglund , and A. Lundberg . 1991. “Lekking in the Black Grouse: A Test of Male Viability.” Nature 352: 155–156. 10.1038/352155a0.

[ece373945-bib-0002] Andersson, M. 1994. Sexual Selection. Princeton University Press.

[ece373945-bib-0004] Björksten, T. A. , K. Fowler , and A. Pomiankowski . 2000. “What Does Sexual Trait FA Tell Us About Stress?” Tree 15: 163–166. 10.1016/S0169-5347(99)01788-7. 10717689

[ece373945-bib-0005] Brooks, M. 2017. “glmmTMB: Generalized Linear Mixed Models using Template Model Builder. R package version 1.1.14.”

[ece373945-bib-0006] Burkhardt, D. 1989. “UV Vision: A Bird's Eye View of Feathers.” Journal of Comparative Physiology A 164: 787–796. 10.1007/BF00616750.

[ece373945-bib-0007] Candolin, U. 2003. “The Use of Multiple Cues in Mate Choice.” Biological Review 78: 575–595. 10.1017/S1464793103006158. 14700392

[ece373945-bib-0008] Catusse, M. 1993. “Spatial and Temporal Plasticity of a Capercaillie ( *Tetrao urogallus* ) Arena in the French Pyrenees.” Ethology Ecology and Evolution 5, no. 2: 145–156. 10.1080/08927014.1993.9523098.

[ece373945-bib-0009] Costanzo, A. , R. Ambrosini , M. Caprioli , et al. 2017. “Lifetime Reproductive Success, Selection on Lifespan, and Multiple Sexual Ornaments in Male European Barn Swallows.” Evolution 71, no. 10: 2457–2468. 10.1111/evo.13312. 28722759

[ece373945-bib-0010] Cuervo, J. J. , and A. P. Møller . 1999. “Phenotypic Variation and Fluctuating Asymmetry in Sexually Dimorphic Feather Ornaments in Relation to Sex and Mating System.” Biological Journal of the Linnean Society 68, no. 4: 505–529. 10.1111/j.1095-8312.1999.tb01186.x.

[ece373945-bib-0011] Cuervo, J. J. , and A. P. Møller . 2000. “Sex‐Limited Expression of Ornamental Feathers in Birds.” Behavioral Ecology 11, no. 3: 246–259. 10.1093/beheco/11.3.246.

[ece373945-bib-0012] Dale, J. 2006. “Intraspecific Variation in Bird Coloration.” In Bird Coloration, edited by G. E. Hill and K. McGraw , 36–86. Harvard University Press.

[ece373945-bib-0013] Delhey, K. , B. Szecsenyi , S. Nakagawa , and A. Peters . 2017. “Conspicuous Plumage Colours Are Highly Variable.” Proceedings of the Royal Society B 284: 20162593. 10.1098/rspb.2016.2593. 28100823 PMC5310046

[ece373945-bib-0014] Fitzpatrick, S. 1998. “Birds' Tails as Signaling Devices: Markings, Shape, Length, and Feather Quality.” American Naturalist 151: 157–173. 10.1086/286109. 18811415

[ece373945-bib-0015] Flor, A. 2023. “Clever Heads Win Fair Ladies. Lekking Observations of a Male Capercaillie Through Seven Consecutive Years.” Grouse News 65: 24–27.

[ece373945-bib-0016] Gibson, R. M. , and J. W. Bradbury . 1987. “Lek Organization in Sage Grouse: Variations on a Territorial Theme.” Auk 104, no. 1: 77–84. 10.2307/4087236.

[ece373945-bib-0017] Hart, N. S. 2001. “Variations in Cone Photoreceptor Abundance and the Visual Ecology of Birds.” Journal of Comparative Physiology. A, Neuroethology, Sensory, Neural, and Behavioral Physiology 187: 685–698. 10.1007/s00359-001-0240-3. 11778831

[ece373945-bib-0019] Hasson, O. 1991. “Sexual Displays as Amplifiers: Practical Examples With an Emphasis on Feather Decorations.” Behavioral Ecology 2: 189–197. 10.1093/beheco/2.3.189.

[ece373945-bib-0020] Hill, G. E. , and J. D. Johnson . 2012. “The Vitamin A‐Redox Hypothesis: A Biochemical Basis for Honest Signaling via Carotenoid Pigmentation.” American Naturalist 180: 127–150. 10.1086/667861. 23070328

[ece373945-bib-0021] Hjorth, I. 1970. “Reproductive Behaviour in Tetraonidae With Special Reference to Males.” Viltrevy, Swedish Wildlife Research 7: 184–587.

[ece373945-bib-0022] Höglund, J. , R. V. Alatalo , R. M. Gibson , and A. Lundberg . 1995. “Mate Choice Copying in Black Grouse.” Animal Behaviour 49: 1627–1633. 10.1016/0003-3472(95)90085-3.

[ece373945-bib-0023] Höglund, J. , R. V. Alatalo , A. Lundberg , and O. Rätti . 1994. “Context‐Dependent Effects of Tail‐Ornament Damage on Mating Success in Black Grouse.” Behavioral Ecology 5, no. 2: 182–187. 10.1093/beheco/5.2.182.

[ece373945-bib-0024] Höglund, J. , M. Eriksson , and L. E. Lindell . 1990. “Females of the Lek‐Breeding Great Snipe, *Gallinago media* , Prefer Males With White Tails.” Animal Behaviour 40, no. 1: 23–32. 10.1016/S0003-3472(05)80662-1.

[ece373945-bib-0025] Jiguet, F. , and V. Bretagnolle . 2014. “Sexy Males and Choosy Females on Exploded Leks: Correlates of Male Attractiveness in the Little Bustard.” Behavioural Processes 103: 246–255. 10.1016/j.beproc.2014.01.008. 24440985

[ece373945-bib-0026] Kose, M. , R. Mänd , and A. P. Møller . 1999. “Sexual Selection for White Tail Spots in the Barn Swallow in Relation to Habitat Choice by Feather Lice.” Animal Behaviour 58, no. 6: 1201–1205. 10.1006/anbe.1999.1249. 10600140

[ece373945-bib-0027] Liu, Y. , E. S. Scordato , R. Safran , and M. Evans . 2018. “Ventral Colour, Not Tail Streamer Length, Is Associated With Seasonal Reproductive Performance in a Chinese Population of Barn Swallows ( *Hirundo rustica gutturalis* ).” Journal of Ornithology 159: 675–685. 10.1007/s10336-018-1555-y.

[ece373945-bib-0028] Loyau, A. , M. S. Jalme , and G. Sorci . 2005. “Intra‐ and Intersexual Selection for Multiple Traits in the Peacock ( *Pavo cristatus* ).” Ethology 111, no. 9: 810–820. 10.1111/j.1439-0310.2005.01091.x.

[ece373945-bib-0029] Lüdecke, D. 2019. “Performance: Assessment of Regression Models Performance. R package version 0.15.3.”

[ece373945-bib-0030] Lumsden, H. G. 1961. “The Display of the Capercaillie.” British Birds 54: 257–272.

[ece373945-bib-0032] McDonald, D. B. 1989. “Correlates of Male Mating Success in a Lekking Bird With Male‐Male Cooperation.” Animal Behaviour 37: 1007–1022. 10.1016/0003-3472(89)90145-0.

[ece373945-bib-0033] McFarlane, M. L. , M. R. Evans , K. A. Feldheim , M. Préault , R. C. Bowie , and M. I. Cherry . 2010. “Long Tails Matter in Sugarbirds—Positively for Extrapair but Negatively for Within‐Pair Fertilization Success.” Behavioral Ecology 21, no. 1: 26–32. 10.1093/beheco/arp147.

[ece373945-bib-0034] Milonoff, M. 1995. “Delayed or Instant Copulation? Effect of Female Copying on Male Mating Decisions.” Wildlife Biology 1, no. 1: 57–61. 10.2981/wlb.1995.001.

[ece373945-bib-0035] Minias, P. , A. Surmacki , K. Kudelska , et al. 2018. “Variation in Melanin Pigmentation of a Sexually Selected Plumage Trait and Its Adaptive Value in the Common Snipe *Gallinago gallinago* .” Ibis 160: 101–111. 10.1111/ibi.12530.

[ece373945-bib-0036] Møller, A. P. 1994. “Male Ornament Size as a Reliable Cue to Enhanced Offspring Viability in the Barn Swallow.” Proceedings of the National Academy of Sciences 91, no. 15: 6929–6932. 10.1073/pnas.91.15.6929. PMC44311 8041723

[ece373945-bib-0037] Møller, A. P. 2021. “Mate Choice, Mating Systems, and Sexual Selection.” In The Behavior of Animals, 2nd Edition: Mechanisms, Function and Evolution, edited by J. J. Bolhuis , L.‐U. Giraldeau , and J. A. Hogan , 315–341. John Wiley & Sons, Ltd. 10.1002/9781119109556.ch12.

[ece373945-bib-0039] Møller, A. P. , and J. Nielsen . 1997. “Differential Predation Cost of a Secondary Sexual Character: Sparrowhawk Predation on Barn Swallows.” Animal Behaviour 54: 1545–1551. 10.1006/anbe.1997.9998. 9794779

[ece373945-bib-0040] Møller, A. P. , and J. P. Swaddle . 1997. Asymmetry, Developmental Stability and Evolution. Oxford University Press.

[ece373945-bib-0041] Møller, A. P. , and R. Thornhill . 1998. “Bilateral Symmetry and Sexual Selection: A Meta‐Analysis.” American Naturalist 151: 174–192. 10.1086/286110. 18811416

[ece373945-bib-0042] Moss, R. 1980. “Why Are Capercaillie Cocks So Big?” British Birds 73: 440–447.

[ece373945-bib-0043] Müller, F. J. 1979. “A 15‐Year Study of a Capercaillie Lek in the Western Röhn‐Mountains (W. Germany).” In Woodland Grouse Symp, edited by T. Lovel , 120–130. WPA.

[ece373945-bib-0044] Pérez‐Rodríguez, L. , R. Jovani , and M. Stevens . 2017. “Shape Matters: Animal Colour Patterns as Signals of Individual Quality.” Proceedings of the Royal Society B: Biological Sciences 284, no. 1849: 2016–2446. 10.1098/rspb.2016.2446. PMC5326527 28228513

[ece373945-bib-0046] Petrie, M. , T. Halliday , and C. Saunders . 1991. “Peahens Prefer Peacocks With Elaborate Trains.” Animal Behaviour 41: 323–331. 10.1016/S0003-3472(05)80484-1.

[ece373945-bib-0047] Pruett‐Jones, S. G. , and M. A. Pruett‐Jones . 1990. “Sexual Selection Through Female Choice in Lawes' Parotia, a Lek‐Mating Bird of Paradise.” Evolution 44, no. 3: 486–501. 10.1111/j.1558-5646.1990.tb05934.x. 28567971

[ece373945-bib-0048] Prum, R. O. 2006. “Anatomy, Physics, and Evolution of Structural Colours.” In Bird Coloration, edited by G. E. Hill and K. McGraw , 295–353. Harvard University Press.

[ece373945-bib-0049] R Development Core Team . 2025. “R: A Language and Environment for Statistical Computing. R Foundation for Statistical Computing.”

[ece373945-bib-0050] Rintamäki, P. T. , R. V. Alatalo , J. Höglund , and A. Lundberg . 1995. “Mate Sampling Behaviour of Black Grouse Females ( *Tetrao tetrix* ).” Behavioral Ecology and Sociobiology 37: 209–215. 10.1007/BF00176719.

[ece373945-bib-0051] Rintamäki, P. T. , J. Höglund , R. V. Alatalo , and A. Lundberg . 2001. “Correlates of Male Mating Success on Black Grouse (*Tetrao tetrix L*.) Leks.” Annales Zoologici Fennici 38: 99–109. http://www.jstor.org/stable/23735755.

[ece373945-bib-0053] Sæther, S. A. , P. Fiske , J. A. Kålås , and J. M. Gjul . 2000. “Females of the Lekking Great Snipe Do Not Prefer Males With Whiter Tails.” Animal Behaviour 59: 273–280. 10.1006/anbe.1999.1301. 10675249

[ece373945-bib-0054] Safran, R. J. , and K. J. McGraw . 2004. “Plumage Coloration, Not Length or Symmetry of Tail‐Streamers, Is a Sexually Selected Trait in North American Barn Swallows.” Behavioral Ecology 15, no. 3: 455–461. 10.1093/beheco/arh035.

[ece373945-bib-0055] Saino, N. , J. J. Cuervo , M. Krivacek , F. de Lope , and A. P. Møller . 1997. “Experimental Manipulation of Tail Ornament Size Affects the Hematocrit of Male Barn Swallows ( *Hirundo rustica* ).” Oecologia 110: 186–190. 10.1007/s004420050148. 28307423

[ece373945-bib-0056] Saino, N. , and A. P. Møller . 1996. “Sexual Ornamentation and Immunocompetence in the Barn Swallow.” Behavioral Ecology 7, no. 2: 227–232. 10.1093/beheco/7.2.227.

[ece373945-bib-0057] Savalli, U. M. 1994. “Tail Length Affects Territory Ownership in the Yellow‐Shouldered Widowbird.” Animal Behavior 48: 105–111. 10.1006/anbe.1994.1216.

[ece373945-bib-0058] Schielzeth, H. 2010. “Simple Means to Improve the Interpretability of Regression Coefficients.” Methods in Ecology and Evolution 1, no. 2: 103–113. 10.1111/j.2041-210X.2010.00012.x.

[ece373945-bib-0059] Siitari, H. , R. V. Alatalo , P. Halme , K. L. Buchanan , and J. Kilpimaa . 2007. “Color Signals in the Black Grouse ( *Tetrao tetrix* ): Signal Properties and Their Condition Dependency.” American Naturalist 169, no. S1: S81–S92. 10.1086/510140. 19426093

[ece373945-bib-0060] Siitari, H. , J. Viitala , and M. Hovi . 2002. “Behavioural Evidence on Ultraviolet Vision in a Tetraonid Species ‐ a Foraging Experiment With Black Grouse ( *Tetrao tetrix* ).” Journal of Avian Biology 33, no. 2: 199–202. 10.1034/j.1600-048X.2002.330212.x.

[ece373945-bib-0061] Simmons, L. W. , J. L. Tomkins , J. S. Kotiaho , and J. Hunt . 1999. “Fluctuating paradigm.” Proceedings of the Royal Society B 266: 593–595. 10.1098/rspb.1999.0677.

[ece373945-bib-0062] Soulsbury, C. D. , M. Matti Kervinen , and C. Christophe Lebigre . 2016. “Curse of the Black Spot: Spotting Negatively Correlates With Fitness in Black Grouse *Lyrurus tetrix* .” Behavioral Ecology 27: 1362–1369. 10.1093/beheco/arw057.

[ece373945-bib-0063] Stein, A. C. , and J. A. C. Uy . 2006. “Plumage Brightness Predicts Male Mating Success in the Lekking Golden‐Collared Manakin, *Manacus vitellinus* .” Behavioral Ecology 17: 41–47. 10.1093/beheco/ari095.

[ece373945-bib-0064] Storch, I. 1997. “Male Territoriality, Female Range Use, and Spatial Organisation of Capercaillie *Tetrao urogallus* Leks.” Wildlife Biology 3: 149–161. 10.2981/wlb.1997.019.

[ece373945-bib-0065] Takahashi, M. , H. Arita , M. Hiraiwa‐Hasegawa , and T. Hasegawa . 2008. “Peahens Do Not Prefer Peacocks With More Elaborate Trains.” Animal Behaviour 75: 1209–1219. 10.1016/j.anbehav.2007.10.004.

[ece373945-bib-0066] Wegge, P. , and B. B. Larsen . 1987. “Spacing of Adult and Subadult Male Common Capercaillie During the Breeding Season.” Auk 104: 481–490. 10.2307/4087547.

[ece373945-bib-0067] Wittzell, H. 1991. “Directional Selection on Morphology in the Pheasant, *Phasianus colchicus* .” Oikos 61: 394–400. 10.2307/3545247.

[ece373945-bib-0069] Zhou, W. , R. T. Kimball , Y. Liu , and S. K. Robinson . 2023. “Functions of Avian Elongated Tails, With Suggestions for Future Studies.” Ibis 165, no. 4: 1091–1106. 10.1111/ibi.13222.

[ece373945-bib-0070] Zuk, M. , R. Thornhill , J. D. Ligon , and K. Johnson . 1990. “Parasites and Mate Choice in Red Jungle Fowl.” American Zoologist 30, no. 2: 235–244. 10.1093/icb/30.2.235.

