# Supplementary material for: A lot of variation and asymmetry in the white patches of male capercaillie tails, but no association with mate choice
Source: Ecol Evol. 2026 Jul 1;16(7):e73945. doi: 10.1002/ece3.73945 (PMC13320634; doi:10.1002/ece3.73945)
Supplement: Supplementary file 1 — Appendix S1: Average coverage of white patches in a tail feather, relative as well as absolute amount of asymmetry in the white patches of tail feather pairs in 10 tail images (estimated visually and measured with ImageJ software) and average symmetry rank of 11 raters for the same tails. See text for more details. [file ECE3-16-e73945-s001.docx]

Appendix

Average coverage of white patches in a tail feather, relative as well as absolute amount of asymmetry in the white patches of tail feather pairs in ten tail images (estimated visually and measured with ImageJ software) and average symmetry rank of 11 raters for the same tails. See text for more details.

| Tail | Average area of white patches in a tail feather (visual estimate) (%) | Average area of white patches in a tail feather (ImageJ) (%) | Relative amount of asymmetry in the white patches of tail feather pairs (visual estimate) | Relative amount of asymmetry in the white patches of tail feather pairs (ImageJ) | Absolute amount of asymmetry in the white patches of tail feather pairs (visual estimate) (pp) | Absolute amount of asymmetry in the white patches of tail feather pairs (ImageJ) (pp) | Evaluators’ average symmetry rank of the tail |
| --- | --- | --- | --- | --- | --- | --- | --- |
|  |  |  |  |  |  |  |  |
|  |  |  |  |  |  |  |  |
| A | 6 | 2 | 0.12 | 0.31 | 1.3 | 1.3 | 4,2 |
| B | 11 | 12 | 0.18 | 0.28 | 3.9 | 6.2 | 4,0 |
| C | 3 | 1 | 0.22 | 0.43 | 1.1 | 0.9 | 5,7 |
| D | 5 | 5 | 0.05 | 0.25 | 0.6 | 2.1 | 2,8 |
| E | 3 | 2 | 0.44 | 0.46 | 3.1 | 1.2 | 7,5 |
| F | 12 | 12 | 0.27 | 0.47 | 6.7 | 11.7 | 9,5 |
| G | 21 | 14 | 0.08 | 0.35 | 3.3 | 11.3 | 4,1 |
| H | 10 | 11 | 0.27 | 0.43 | 5.6 | 9.0 | 9,2 |
| I | 13 | 12 | 0.10 | 0.27 | 2.8 | 5.6 | 5,2 |
| J | 8 | 8 | 0.11 | 0.27 | 1.7 | 4.2 | 2,9 |
|  |  |  |  |  |  |  |  |
